# Physiological and Perceptual Internal Load During Kitesurfing Under Real-World Sea Conditions

**DOI:** 10.3390/sports14030117

**Published:** 2026-03-17

**Authors:** Nicola Mancini, Nicola Mangione, Siria Mancini, Vlad Teodor Grosu, Emilia Florina Grosu, Mariasole Antonietta Guerriero, Dan Monea, Giovanni Messina, Marcellino Monda, Rita Polito, Fiorenzo Moscatelli

**Affiliations:** 1 Department of Education and Sport Sciences, Pegaso Telematic University, 80143 Naples, Italy; nicola.mancini@unipegaso.it; 2 Department of Experimental and Clinical Medicine, University of Foggia, 71121 Foggia, Italy; 3 Department of Mechatronics and Machine Dynamics, Universitatea Tehnica, 400114 Cluj-Napoca, Romania; 4 Doctoral School of Physical Education and Sport, Babeș-Bolyai University, 400347 Cluj-Napoca, Romania; emilia.grosu@ubbcluj.ro; 5 Department of Humanistic Studies, University of Foggia, 71121 Foggia, Italy; mariasole.guerriero@unifg.it; 6 Department of Sports Games, Babes-Bolyai University, 400347 Cluj-Napoca, Romania; 7 Department of Experimental Medicine, Section of Human Physiology and Unit of Dietetics and Sports Medicine, University of Campania “Luigi Vanvitelli”, 80138 Naples, Italy; 8 Department of Psychology and Health Sciences, Pegaso Telematic University, 80143 Naples, Italy

**Keywords:** kitesurfing, heart rate, internal load, session rating of perceived exertion, global navigation satellite system

## Abstract

***Background:*** Kitesurfing is a wind-propelled water sport performed in highly variable environmental conditions. Scientific evidence describing internal load under standardized ecological sea constraints remains limited. ***Aim:*** This study aimed to characterize cardiovascular and perceptual responses during a standardized kitesurfing session and to examine associations among heart rate-based internal load indices, session rating of perceived exertion, and global navigation satellite system-derived external output variables. ***Methods:*** A total of 112 male recreational kitesurfers (32.1 ± 6.8 years) completed a 40–50 min standardized session under monitored wind conditions (17–22 knots) along a predefined approximately 800 m course. Heart rate was continuously recorded, and session rating of perceived exertion (Borg Category-Ratio 10 scale) was collected 30 ± 5 min post-session. Training impulse, mean percentage of maximal heart rate, and session rating of perceived exertion load were calculated. Pearson correlation analyses with bootstrapping (1000 resamples) and five percent trimming were performed, with statistical significance set at 0.05. ***Results:*** Sessions were performed at 78.4 ± 9.1 percent of maximal heart rate. Training impulse and mean percentage of maximal heart rate were strongly associated (correlation coefficient = 0.90, probability value < 0.001), reflecting the shared heart rate-based structure of these metrics. Training impulse showed a moderate association with session rating of perceived exertion load (correlation coefficient = 0.46, probability value < 0.001). No significant associations were observed between internal load indices and global navigation satellite system-derived mean speed (correlation coefficient = −0.14, probability value = 0.149) or distance (correlation coefficient = 0.06, probability value = 0.555). Sensitivity analyses confirmed the stability of the observed associations. ***Conclusions:*** Under standardized ecological sea conditions, kitesurfing sessions were characterized by sustained high submaximal cardiovascular intensity. Heart rate-based and perceptual measures showed consistent associations within this protocol, whereas global navigation satellite system-derived external outputs were not significantly related to internal load indices. Within the limits of this cross-sectional ecological design, the combined use of one heart rate-based indicator and session rating of perceived exertion offers a coherent and practically interpretable description of session internal load in open-water kitesurfing.

## 1. Introduction

Kitesurfing (or kiteboarding) is a wind-propelled water sport that has expanded rapidly since the late 1990s, combining elements of surfing, windsurfing, and traction kiting. The athlete controls a kite via a four-line bar system and converts wind energy into propulsion on a small board, adopting variable postures according to environmental and technical demands. Recognized disciplines include freestyle, speed/slalom, wave riding, and course racing, the latter aligned with Olympic sailing formats [1,2]. From a physiological perspective, current knowledge is largely extrapolated from related sail-propelled sports such as windsurfing and dinghy sailing, where energetic demands have been characterized using direct measurements of oxygen uptake [3,4,5,6,7,8,9,10]. These sports typically involve a mixed physiological profile combining dynamic exercise with sustained isometric or quasi-isometric efforts, particularly during hiking posture, with substantial involvement of the lower and upper limbs [5,6,11,12,13]. Kitesurfing shares several of these features, including intermittent accelerations and prolonged stabilization demands, while adding sport-specific requirements related to kite handling and whole-body balance under variable wind and sea conditions. Direct assessment of metabolic demand in real-sea kitesurfing is logistically challenging due to spray, wind gusts, and movement instability, although portable metabolic systems have shown acceptable validity in dynamic field settings [14]. Within contemporary training theory, internal load reflects the individual physiological and perceptual response to an external stimulus, whereas external output describes the mechanical work performed [10]. In applied contexts, heart rate is widely used as a proxy for exercise intensity and internal load [15,16], whereas blood lactate provides complementary information on metabolic contribution [9,17]. In outdoor aquatic sports, external output is commonly estimated through global positioning system (GPS)-derived variables such as speed and displacement [18]. However, in environments strongly influenced by wind and water conditions, mechanical displacement does not necessarily correspond to physiological strain. Integrating heart rate-based metrics with perceptual indicators may therefore provide a more coherent description of internal load under ecological conditions characterized by environmental variability [19]. Physiological research specifically focused on kitesurfing remains limited. Although injury and safety aspects have been addressed [20,21,22,23,24,25], relatively few investigations have described cardiometabolic responses during open-water practice. Previous freestyle trials and light-wind tasks have reported mean heart rate values corresponding to approximately 80–85% of maximal heart rate and post-exercise lactate concentrations around 5 mmol·L^−1^ [26,27]. Nevertheless, large datasets collected under meteorologically restricted and technically standardized sea conditions, combining heart rate, perceptual responses, and GPS-derived external outputs, remain scarce. To address this gap, the present study adopted an ecological field design in which wind conditions were monitored and restricted to a predefined operational range, while course geometry and equipment were standardized. Internal load was described using a heart rate-based index (training impulse) and session rating of perceived exertion [28,29], representing complementary dimensions of physiological intensity and perceived global strain. For contextual purposes, a heart rate-derived estimate of maximal oxygen uptake was also reported with appropriate interpretative caution [30]. The aim of this study was to characterize cardiovascular and perceptual responses during a standardized kitesurfing session under real-world sea conditions and to examine the associations among training impulse, mean percentage of maximal heart rate, session perceived exertion, and GPS-derived external output variables (mean speed and distance). We hypothesized that: (i) training impulse would show a strong association with mean percentage of maximal heart rate, given their shared heart rate-based structure; (ii) training impulse would be moderately associated with session perceived exertion; and (iii) associations between internal load indices and GPS-derived external output variables would be weak or non-significant under standardized sea conditions.

## 2. Materials and Methods

### 2.1. Study Design

An observational cross-sectional design was adopted in a natural outdoor environment. Data collection was conducted at two coastal locations in Southern Italy (Lesina and Marsala, Italy) in collaboration with local kitesurfing schools.

Sessions were performed between June and July 2025, within the time window of 10:30–16:30, to limit microclimatic variability. Wind conditions were monitored and restricted to a predefined operational range (17–22 knots) under the side or side–onshore direction.

### 2.2. Participants

A total of 129 male recreational kitesurfers (≥2 sessions·week^−1^) were recruited. Seventeen participants were excluded (heart rate signal loss, *n* = 6; meteorological conditions outside inclusion range, *n* = 7; incomplete protocol completion, *n* = 4), resulting in a final sample of 112 participants.

Inclusion criteria were: age 18–50 years; ≥1 year of kitesurfing experience; valid medical clearance; absence of known cardiovascular disease. Exclusion criteria included stimulant intake within 24 h, history of syncope, or inability to complete the protocol.

All participants provided written informed consent. The study was conducted in accordance with the Declaration of Helsinki and approved by the Institutional Ethics Committee of Pegaso Telematic University (PROT/E 002466, 29 March 2024).

### 2.3. Session Standardization

Each athlete completed 40–50 min of continuous riding along straight segments (~800 m) delimited by buoys, alternating right and left tack. One controlled jump per segment (front take-off and landing without rotations) was permitted to maintain technical consistency.

**Figure 1:**
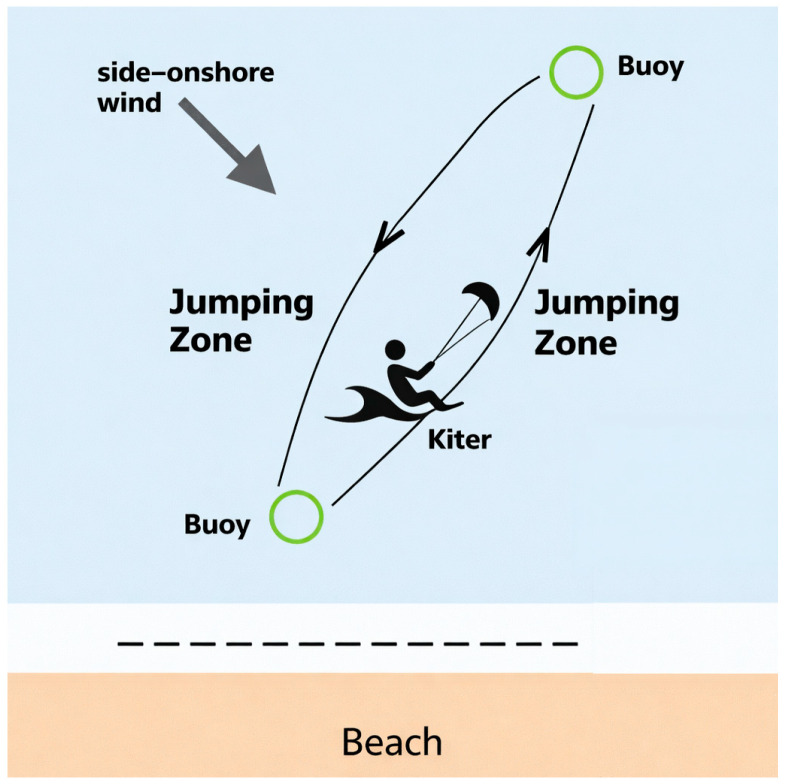
Schematic representation of the experimental protocol under side–onshore conditions: two buoys spaced ~800 m apart, upwind/downwind legs, and one jump per leg. Target duration: 40–50 min; wind range: 17–22 kn; continuous HR and GPS monitoring. The dotted line indicates the shoreline separating the sea area from the beach.

The target workload was 9–10 km. Effective riding duration was required to be ≥85% of the planned session time. Compliance with route geometry and duration was verified via GPS tracking and shore-based observation (Figure 1).

### 2.4. Instrumentation and Measurements

#### 2.4.1. Heart Rate (HR)

Heart rate was recorded using a Polar H10 chest strap (Polar Electro Oy, Kempele, Finland), previously validated against electrocardiography for R–R interval detection. Data were sampled at 1 Hz and recorded via Polar Flow.

#### 2.4.2. Blood Lactate

Capillary blood lactate was measured using a portable analyzer (Lactate Scout, EKF Diagnostics, Leipzig, Germany). Samples were obtained at rest, immediately post-session, and after 60 s of passive recovery. Lactate values were collected to provide a descriptive metabolic characterization of the session and to contextualize the cardiovascular and perceptual responses observed. These values were used descriptively and were not included in the correlational analyses.

#### 2.4.3. GPS and External Output

External output variables were recorded using a Polar M430 watch (Polar Electro Oy, Kempele, Finland; 1 Hz). Data were exported in .csv format and synchronized with heart rate data. To verify positional accuracy, 20 sessions were additionally recorded using a Garmin GLO 2 GNSS receiver (Garmin Ltd., Olathe, KS, USA; 10 Hz). Only tracks with 3D fix ≥ 95% and HDOP ≤ 2.0 were retained. Agreement between devices was high (r > 0.95) for distance and mean speed. However, this cross-validation procedure does not permit precise quantification of absolute positional or speed measurement uncertainty. The Polar M430 was selected due to its widespread use in applied sport settings, robustness in field conditions, and compatibility with synchronized heart rate recording. Although higher-frequency and research-grade GNSS devices are available, the present study prioritized ecological feasibility and integration within routine beach practice. The M430 supports multi-satellite reception (GPS/GLONASS), which improves signal stability compared with single-system receivers; however, like all wrist-based GNSS devices, it remains subject to typical positional uncertainty under dynamic outdoor conditions. For this reason, only tracks meeting predefined HDOP and signal-fix criteria were retained. GPS-derived variables reflect displacement and speed relative to ground position.

#### 2.4.4. Meteorological Monitoring

Wind speed (mean and gust) and air temperature were measured using Bluetooth anemometers (Kestrel 5500, Nielsen-Kellerman, Boothwyn, PA, USA; or Skywatch BL, JDC Electronic SA, Yverdon-les-Bains, Switzerland). Data were averaged over 10 min intervals. Anemometers were calibrated against a fixed harbor meteorological station (r^2^ > 0.95). The fixed station provided reference data for inclusion criteria; portable devices were used for operational monitoring.

#### 2.4.5. Sport Equipment

**Figure 2:**
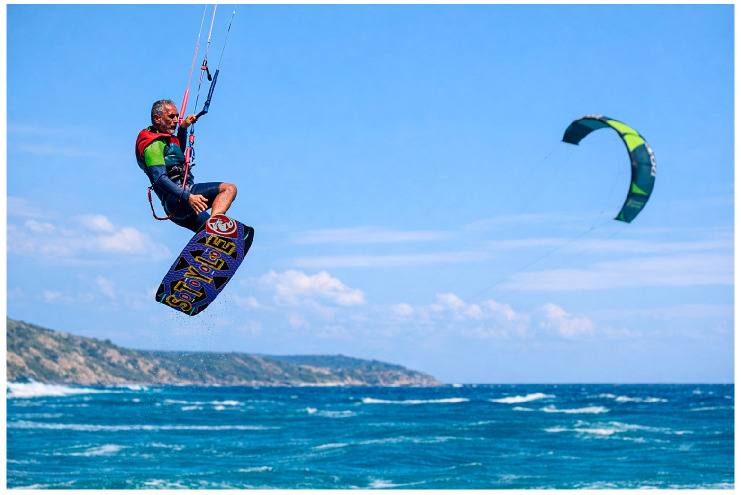
Example of standardized equipment configuration used during data collection (freeride kite, twin-tip board, harness, wetsuit). © the authors.

Participants used 10–12 m^2^ kites (freeride/all-around, four-line models), twin-tip boards ranging from 136 × 38–40 cm to 140 × 41–42 cm, harnesses, and wetsuits (2–3 mm) (Figure 2). Equipment setup was checked prior to each session for integrity, inflation pressure, and line symmetry.

### 2.5. Meteorological and Environmental Inclusion Criteria

Sessions were included only if the following environmental criteria were met: mean wind speed between 17 and 22 knots, gust factor (gust/mean) < 1.25, intra-session wind variation ≤ ±10%, wind direction within ±20° of the spot axis, significant wave height < 0.7 m, and air temperature between 22 and 30 °C. Meteorological data were synchronized with physiological data using timestamps and averaged over a 10 min window centered on session mid-time.

### 2.6. Internal Load and Perceptual Measures

Resting heart rate (HRrest) was recorded in the morning in supine position after at least 10 min of quiet rest. Maximal heart rate (HRmax) was obtained during a Yo-Yo Endurance Test (Level 1) performed 7 days prior under standardized conditions.

Mean percentage of maximal heart rate was calculated as follows:

%HRmax_mean = 100 × HRmean/HRmax

Training impulse (TRIMP) was calculated using a linear Banister formulation [31]:

TRIMP = t × (HRmean − HRrest)/(HRmax − HRrest)

where t represents session duration (minutes).

Session rating of perceived exertion was assessed using the Borg Category-Ratio scale (CR-10; 0–10), administered 30 ± 5 min after session completion in a quiet area, without peer influence. The standardized script was as follows: “Considering the entire session, how intense was it overall? (0 = rest; 10 = maximal).”

Session perceived load was calculated as follows [28,29]:

sRPE = RPE × session duration (minutes).

Data were collected individually by the same trained operator whenever possible.

All RPE assessments were performed individually and blinded to HR and GPS data to prevent peer comparison or anchoring bias. Adherence to the predefined timing window was ≥95%. Cases outside this window (*n* = 5) were excluded from sRPE-related correlational analyses.

Maximal oxygen uptake (VO_2_max) was estimated using the heart rate ratio method [30] and interpreted as an indirect indicator of aerobic capacity. This variable was included for descriptive contextualization of the sample’s aerobic profile and was not considered a primary outcome of the study.

### 2.7. Data Processing and Quality Control

Data were exported from Polar Flow and merged with meteorological logs. Quality control included exclusion of HR signal loss > 20%, verification of physiological plausibility (HR_rest < HR_mean; %HRmax_mean ≤ 100%), and conservative outlier screening (±3 SD). Missing data (<2%) were handled by listwise exclusion for the specific analysis.

### 2.8. Statistical Analysis

Statistical analyses were conducted using IBM SPSS Statistics v25 (IBM Corp., Armonk, NY, USA) and JASP v0.18 (University of Amsterdam, Amstersam, The Netherlands). Continuous variables are presented as mean ± SD. Normality was assessed using the Shapiro–Wilk test. Pearson correlations were computed for predefined associations between internal load indices (TRIMP, %HRmax_mean, sRPE) and external output variables (mean speed, distance), as well as between physiological and perceptual measures. Robustness was evaluated using bootstrapping (1000 resamples) with 95% confidence intervals and 5% trimming. Statistical significance was set at *p* < 0.05 (two-tailed). The magnitude of correlation coefficients was interpreted according to commonly used thresholds in sport and exercise science: trivial (<0.10), small (0.10–0.29), moderate (0.30–0.49), large (0.50–0.69), very large (0.70–0.89), and nearly perfect (≥0.90) [32].

## 3. Results

### 3.1. Sample Characteristics

The analyzed sample comprised 112 male kitesurfers (age: 32.1 ± 6.8 years; height: 176.2 ± 4.2 cm; body mass: 72.1 ± 7.0 kg; experience: 4.2 ± 1.7 years). Mean session duration was 45.4 ± 3.0 min. Mean heart rate during the standardized session was 137.5 ± 16.6 bpm. The reference maximal heart rate determined during the Yo-Yo Endurance Test was 175.9 ± 13.2 bpm, corresponding to a mean relative intensity of 78.4 ± 9.1% HRmax.

Mean internal load indices were: TRIMP 28.8 ± 7.3 AU; RPE 6.7 ± 1.1; sRPE 301.9 ± 50.5 AU. GPS-derived outputs included mean speed 19.6 ± 1.7 km·h^−1^, maximal speed 26.9 ± 4.3 km·h^−1^, and total distance 14.8 ± 1.7 km. Mean wind speed during sessions was 19.6 ± 2.4 kn and air temperature was 27.1 ± 2.3 °C.

Blood lactate increased from 2.2 ± 0.4 mmol·L^−1^ at baseline to 3.8 ± 0.6 mmol·L^−1^ immediately post-session and decreased to 2.9 ± 0.3 mmol·L^−1^ after 60 s of recovery.

A complete summary of variables is provided in Table 1.

### 3.2. Associations Between Internal Load, Perceptual Measures, and External Output

Pearson’s correlation coefficients were computed to examine associations among internal load indices, perceptual measures, and external output variables (*n* = 112; α = 0.05), following confirmation of approximate normality.

#### 3.2.1. Internal Load

A weak positive correlation was observed between sRPE and session duration (r = 0.24, *p* = 0.010). sRPE was strongly correlated with RPE (r = 0.93, *p* < 0.001), reflecting the mathematical structure of the index. TRIMP showed a very strong correlation with %HRmax_mean (r = 0.90, *p* < 0.001) and a moderate correlation with sRPE (r = 0.46, *p* < 0.001). A weak-to-moderate association was also found between TRIMP and session duration (r = 0.28, *p* = 0.003).

#### 3.2.2. External Output

**Figure 3:**
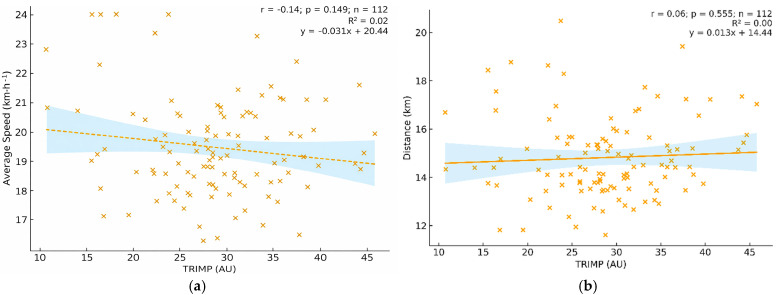
Associations between internal load and external output variables (*n* = 112). (**a**) Mean speed (km·h^−1^) as a function of TRIMP (AU) (r = −0.14, *p* = 0.149). (**b**) Total distance covered (km) as a function of TRIMP (AU) (r = 0.06, *p* = 0.555). Points represent individual participants; the yellow dotted/solid line represents the linear regression fitted to the data points, illustrating the overall trend between TRIMP and the external output variable, and the light blue shaded area represents the 95% confidence interval.

No significant correlations were observed between TRIMP and mean speed (r = −0.14, *p* = 0.149) or total distance covered (r = 0.06, *p* = 0.555) (Figure 3).

**Figure 4:**
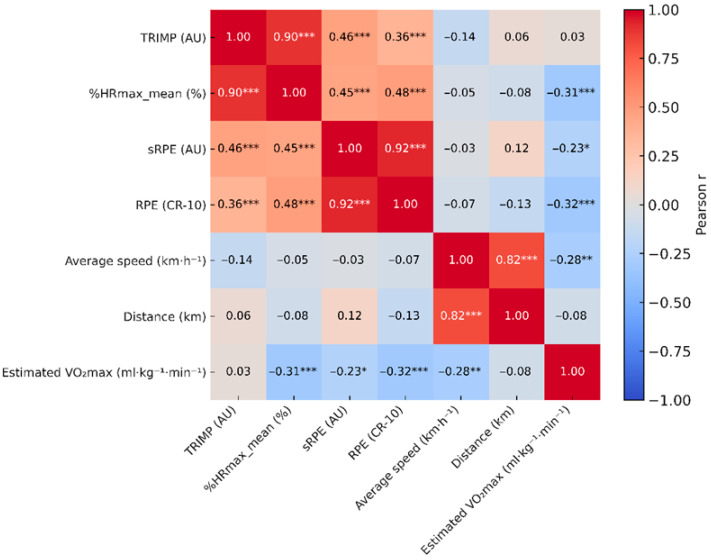
Pearson correlation matrix (r) among physiological indices (TRIMP, %HRmax_mean, HR_rest, HR_max, estimated VO_2_max), perceptual measures (RPE, sRPE), and external output variables (mean speed, distance) (*n* = 112). Asterisks indicate statistical significance (* *p* < 0.05; ** *p* < 0.01; *** *p* < 0.001). Color scale represents direction and magnitude of correlations (−1 to +1).

The overall correlation structure among physiological, perceptual, and external variables is summarized in Figure 4.

### 3.3. Estimated Aerobic Capacity

Estimated VO_2_max was inversely correlated with HR_rest (r = −0.55, *p* < 0.001) and positively correlated with HR_max (r = 0.42, *p* < 0.001). Bootstrapped 95% confidence intervals confirmed the stability of these associations.

## 4. Discussion

### 4.1. Interpretation of Findings

The present study examined physiological and perceptual internal load during a standardized kitesurfing session performed under standardized ecological sea conditions. The main findings indicate a strong association between heart rate-based internal load indices and relative cardiac intensity, a moderate convergence between physiological and perceptual markers, and weak or absent associations between internal load and GPS-derived external outputs. The observed relative intensity indicates that the standardized session was performed within a sustained high submaximal cardiovascular range under the predefined environmental constraints. Post-session lactate values were reported for descriptive purposes only; no formal threshold-based classification of exercise intensity domains was performed, and metabolic interpretation should therefore be made with caution. This physiological pattern is broadly consistent with previous freestyle investigations reporting high submaximal cardiovascular demands and moderate lactate responses during competitive heats [26,33].

Importantly, the strong alignment between TRIMP and mean relative heart rate confirms internal coherence within HR-derived indices. The moderate association between TRIMP and sRPE supports convergent validity between objective and perceptual markers of internal load, in line with field-based monitoring frameworks previously described in outdoor and team sports contexts [28,29].

### 4.2. Convergence and Complementarity of TRIMP and sRPE

TRIMP integrates relative cardiac stimulus over time, whereas sRPE represents a global perceptual evaluation of the session [28,29]. In the present data, the close alignment between TRIMP and relative heart rate confirms the expected mathematical and physiological relationship between HR-based indices. However, the moderate association between TRIMP and sRPE suggests partial overlap rather than redundancy.

This pattern indicates that perceptual responses likely integrate additional components not fully captured by mean heart rate alone, including mechanical stress (e.g., landings, board–water interaction, vibration), attentional demand, and postural stabilization in unstable conditions. Operationally, this supports the complementary use of one HR-based indicator (TRIMP or %HRmax_mean) together with sRPE when monitoring ecological kitesurfing sessions.

### 4.3. Internal Load and External Output: Evidence of Partial Decoupling

No meaningful associations were observed between internal load indices and GPS-derived measures of mean speed or total distance. This lack of association indicates that, under standardized wind constraints, GPS-derived speed and distance did not systematically covary with internal load indices in the present dataset. These findings suggest that GPS-derived outputs should not be interpreted as direct surrogates of physiological stress but rather as context-dependent mechanical indicators.

### 4.4. Aerobic Profile and Cardiac Gradient

Estimated aerobic capacity showed expected associations with resting and maximal heart rate, consistent with established physiological relationships. Individuals with lower resting heart rate tended to display higher estimated aerobic capacity.

However, VO_2_max in the present study was derived using a heart rate ratio method [30], which entails mathematical coupling with cardiac variables. For this reason, analyses primarily focused on relative heart rate during the session as the indicator of exercise intensity. Associations involving estimated VO_2_max should therefore be interpreted as indirect trends rather than direct metabolic evidence [28,29].

Overall, the physiological profile observed was compatible with sustained high submaximal cardiovascular intensity within the standardized protocol adopted.

### 4.5. Implications for Monitoring in Open-Water Contexts

The findings indicate that, within the standardized ecological framework adopted, the combination of one HR-based indicator (TRIMP or %HRmax_mean) and one perceptual measure (sRPE) provides a consistent representation of internal load.

This dual approach reflects both relative cardiac intensity and global perceived strain within the standardized session framework. GPS-derived measures such as speed and distance remain useful for technical and contextual analysis but should not be interpreted as direct indicators of physiological stress. Their interpretation requires consideration of wind intensity, gust variability, and course geometry.

Standardized administration of the Borg CR-10 scale approximately 30 min post-session enhances comparability across athletes and sessions in ecological settings [28,29].

### 4.6. Strengths and Limitations

The study presents several strengths, including ecological standardization of environmental conditions, a relatively large sample size for this discipline, multimodal integration of physiological and perceptual measures, and the use of robustness procedures (bootstrapping and trimming) to verify the stability of the main associations.

Several limitations should be acknowledged. First, no direct in-water metabolic measurements were performed, and maximal oxygen uptake was estimated using a heart rate-based ratio method [30], which entails inherent mathematical coupling with cardiac variables.

In addition, maximal heart rate was determined using a terrestrial Yo-Yo Endurance Test (Level 1). Although this procedure provided a standardized reference across participants, it may not fully reflect the sport-specific maximal cardiovascular response of kitesurfing, a discipline characterized by intermittent effort patterns, sustained isometric components, and substantial upper-body stabilization demands. Consequently, relative intensity indices (%HRmax_mean) and TRIMP values should be interpreted as standardized reference measures rather than sport-specific maximal cardiovascular indicators.

Further methodological caution concerns GNSS-derived external output variables. GPS measures displacement and speed relative to ground position and does not account for water currents acting beneath the athlete. In aquatic environments, underlying current flow may influence recorded speed and distance independently of actual propulsive effort or technical execution. Although environmental inclusion criteria and wind-range restrictions were applied to reduce variability, residual current effects cannot be entirely excluded. The additional verification performed using a Garmin GLO 2 receiver demonstrated high agreement (r > 0.95) for distance and mean speed; however, this cross-validation procedure does not allow precise quantification of absolute positional or speed measurement uncertainty.

Recent investigations on consumer multi-constellation GNSS devices have reported typical positional errors ranging from approximately 1 to 5 m under open-sky conditions, with velocity accuracy influenced by sampling frequency, satellite geometry (HDOP), multipath effects, and rapid changes in acceleration [34]. Devices operating at 1 Hz, particularly wrist-worn sport watches, may exhibit increased variability during highly dynamic activities compared with higher-frequency (5–10 Hz) systems. Accordingly, although multi-satellite reception (GPS/GLONASS) enhances signal robustness, GNSS-derived speed and displacement values should be interpreted within the expected measurement uncertainty of consumer-grade devices rather than as laboratory-grade kinematic outputs.

Therefore, GNSS-derived variables should be interpreted as contextual mechanical descriptors rather than direct performance indicators.

Finally, the sample consisted exclusively of male recreational kitesurfers, which limits generalizability to female athletes and other performance levels.

### 4.7. Future Directions

Future investigations should incorporate direct metabolic measurements during open-water sessions to refine intensity classification. The integration of high-frequency multi-constellation GNSS systems and environmental variability indices may clarify the proportion of mechanical output variance not explained by internal load. Longitudinal and test–retest designs are also warranted to evaluate the reliability and responsiveness of HR-based and perceptual monitoring tools in real-sea conditions.

## 5. Conclusions

Under standardized ecological wind and sea constraints, the kitesurfing sessions examined were performed at a sustained high submaximal cardiovascular intensity. Heart rate-based internal load indices demonstrated strong internal coherence and moderate convergence with perceptual measures, indicating consistent associations between physiological and subjective markers within this standardized protocol. GPS-derived external output variables were not significantly associated with internal load indices in the present dataset, suggesting that displacement and speed over ground do not systematically reflect cardiovascular strain under monitored sea conditions. These external measures should therefore be interpreted as contextual mechanical descriptors rather than direct indicators of physiological load. Estimated aerobic capacity showed expected associations with resting and maximal heart rate; however, given its indirect derivation from cardiac variables and the use of a terrestrial HR_max assessment, these values should be interpreted cautiously. Within the constraints of the present cross-sectional ecological design, the combined use of one HR-based indicator (TRIMP or %HRmax_mean) together with session rating of perceived exertion provides a coherent and practically interpretable framework for monitoring internal load during standardized open-water kitesurfing sessions.

## Figures and Tables

**Table 1:** Physiological, performance, and environmental variables (*n* = 112) (values expressed as M ± SD).

Variable	M ± SD	Min–Max	Unit
Age	32.1 ± 6.8	22–50	years
Height	176.2 ± 4.2	166–184	cm
Body mass	72.1 ± 7.0	58–90	kg
Experience	4.2 ± 1.7	1–9	years
HR_rest	71.6 ± 10.4	51–93	bpm
HR_mean	137.5 ± 16.6	98–172	bpm
HR_max	175.9 ± 13.2	157–205	bpm
%HRmax_mean	78.4 ± 9.1	60.1–95.3	%
TRIMP	28.8 ± 7.3	10.7–45.8	AU
VO_2_max	38.5 ± 6.9	27.8–56.1	ml·kg^−1^·min^−1^
Duration	45.4 ± 3.0	38.4–52.0	min
Mean speed	19.6 ± 1.7	16.4–24.0	km·h^−1^
Maximal speed	26.9 ± 4.3	25.0–40.4	km·h^−1^
Distance	14.8 ± 1.7	11.6–20.5	km
RPE (0–10)	6.7 ± 1.1	4–8	Borg CR-10 scale
sRPE	301.9 ± 50.5	191.0–396.6	AU
Wind speed	19.6 ± 2.4	17.0–22.0	kn

Note: RPE = rating of perceived exertion (Borg Category-Ratio 10 scale, CR-10, 0–10), assessed 30 min post-session; sRPE (arbitrary units, AUs) = RPE × duration (min). Meteorological data were obtained from the local anemometric station and summarized over a 10 min window centered on the mid-time of the session, in accordance with the inclusion criteria (mean wind 17–22 kn; air temperature 22–30 °C). HR_max was determined using the Yo-Yo Endurance Test (Level 1) performed 7 days prior to the on-water session and defined as the test HR_peak.

## Data Availability

The data presented in this study are available in the article.

## Abbreviations

The following abbreviations are used in this manuscript:

HR	Heart Rate
HR_rest	Resting Heart Rate
HR_mean	Mean Heart Rate
HR_max	Maximal Heart Rate
%HRmax_mean	Mean Percentage of Maximal Heart Rate
TRIMP	Training Impulse
RPE	Rating of Perceived Exertion
sRPE	Session Rating of Perceived Exertion
VO_2_	Oxygen Uptake
VO_2_max	Maximal Oxygen Uptake
GPS	Global Positioning System
GNSS	Global Navigation Satellite System
HDOP	Horizontal Dilution of Precision
CR-10	Borg Category-Ratio 10 Scale
AUs	Arbitrary Units
ECG	Electrocardiography
CI	Confidence Interval

## References

[1] International Kiteboarding Association (IKA) (2023). Discovery, Intermediate, Independent, Advanced, Evolution, Safety and Instructor Handbooks.

[2] Journal of Sports Sciences Editorial Office (2007). Glossary of Sailing Terms. J. Sports Sci..

[3] De Vito G., Di Filippo L., Felici F., Gallozzi C., Madaffari A., Marino S. (1996). Assessment of energetic cost in Laser and Mistral sailors. Int. J. Sport Cardiol..

[4] De Vito G., Di Filippo L., Rodio A., Felici F., Madaffari A. (1997). Is the Olympic boardsailor an endurance athlete?. Int. J. Sports Med..

[5] Vogiatzis I., Roach N. (1993). Cardiovascular, muscular and lactate responses during sailing. Med. Sci. Res..

[6] Vogiatzis I., Spurway N.C., Jennett S., Wilson J., Sinclair J. (1996). Changes in ventilation related to changes in electromyograph activity during repetitive bouts of isometric exercise in simulated sailing. Eur. J. Appl. Physiol. Occup. Physiol..

[7] Castagna O., Brisswalter J. (2007). Assessment of energy demand in Laser sailing. Eur. J. Appl. Physiol..

[8] Castagna O., Vaz Pardal C., Brisswalter J. (2007). The assessment of energy demand in the new Olympic windsurf board. Eur. J. Appl. Physiol..

[9] Guével A., Maïsetti O., Prou E., Dubois J.J., Marini J.F. (1999). Heart rate and blood lactate responses during sailing. J. Sports Sci..

[10] Chamari K., Moussa-Chamari I., Galy O., Chaouachi M., Koubaa D., Ben Hassen C., Hue O. (2003). Correlation between heart rate and performance during Olympic windsurfing. Eur. J. Appl. Physiol..

[11] Felici F., Rodio A., Madaffari A., Ercolani L., Marchetti M. (1999). The cardiovascular work of competitive dinghy sailing. J. Sports Med. Phys. Fitness.

[12] Spurway N.C. (2007). Hiking physiology and the “quasi-isometric” concept. J. Sports Sci..

[13] Vogiatzis I., Tzineris D., Athanasopoulos D., Georgiadou O., Geladas N. (2008). Quadriceps oxygenation during isometric exercise in sailing. Int. J. Sports Med..

[14] McLaughlin J.E., King G.A., Howley E.T., Bassett D.R., Ainsworth B.E. (2001). Validation of the COSMED K4b^2^ portable metabolic system. Int. J. Sports Med..

[15] Bernard T., Gavarry O., Bermon S., Giacomoni M., Marconnet P., Falgairette G. (1997). Relationships between oxygen consumption and heart rate. Eur. J. Appl. Physiol..

[16] Keytel L.R., Goedecke J.H., Noakes T.D., Hiiloskorpi H., Laukkanen R., van der Merwe L., Lambert E.V. (2005). Prediction of energy expenditure from heart rate monitoring during submaximal exercise. J. Sports Sci..

[17] Pyne D.B., Boston T., Martin D.T., Logan A. (2000). Evaluation of the Lactate Pro blood lactate analyser. Eur. J. Appl. Physiol..

[18] Witte T.H., Wilson A.M. (2004). Accuracy of non-differential GPS for the measurement of speed. J. Biomech..

[19] Farley O.R.L., Abbiss C.R., Sheppard J.M. (2017). Performance analysis of surfing: A review. J. Strength Cond. Res..

[20] Petersen W., Hansen U., Zernial O., Nickel C., Prymka M. (2002). Mechanisms and prevention of kitesurfing injuries. Sportverletz. Sportschaden.

[21] Petersen W., Nickel C., Zantop T., Zernial O. (2005). Kitesurfing injuries. Orthopäde.

[22] Nickel C., Zernial O., Musahl V., Hansen U., Zantop T., Petersen W. (2004). A prospective study of kitesurfing injuries. Am. J. Sports Med..

[23] Exadaktylos A.K., Sclabas G.M., Blake I., Swemmer K., McCormick G., Erasmus P. (2005). The kick with the kite. Br. J. Sports Med..

[24] Lundgren L., Brorsson S., Osvalder A. (2012). Comfort aspects important for kitesurfing. Work.

[25] Bourgois J.G., Boone J., Callewaert M., Tipton M.J., Tallir I.B. (2014). Biomechanical and physiological demands of kitesurfing. Sports Med..

[26] Camps A., Vercruyssen F., Brisswalter J. (2011). Heart rate and blood lactate concentration changes in kitesurfing. J. Sports Med. Phys. Fitness.

[27] Vercruyssen F., Blin N., L’Huillier D., Brisswalter J. (2009). Assessment of physiological demand in kitesurfing. Eur. J. Appl. Physiol..

[28] Foster C. (2001). A new approach to monitoring exercise training. Med. Sci. Sports Exerc..

[29] Impellizzeri F.M., Rampinini E., Coutts A.J., Sassi A., Marcora S.M. (2004). Use of RPE-based training load in soccer. Med. Sci. Sports Exerc..

[30] Uth N., Sørensen H., Overgaard K., Pedersen P.K. (2004). Estimation of VO_2_max from the HRmax/HRrest ratio. Eur. J. Appl. Physiol..

[31] Banister E.W., MacDougall J.D., Wenger H.A., Green H.J. (1991). Modeling elite athletic performance. Physiological Testing of the High-Performance Athlete.

[32] Hopkins W.G., Marshall S.W., Batterham A.M., Hanin J. (2009). Progressive statistics for studies in sports medicine and exercise science. Med. Sci. Sports Exerc..

[33] Vercruyssen F., Brisswalter J., Hausswirth C., Bernard T. (2008). Assessment of the physiological demand during kitesurfing. Eur. J. Appl. Physiol..

[34] Jacko T., Bartsch J., von Diecken C., Ueberschär O. (2024). Validity of Current Smartwatches for Triathlon Training: How Accurate Are Heart Rate, Distance, and Swimming Readings?. Sensors.

